# Dust mitigation by the application of treated sewage effluent (TSE) in Iran

**DOI:** 10.1038/s41598-022-19331-0

**Published:** 2022-09-15

**Authors:** Maryam Naeimi, Alireza Eftekhari, Rostam Khalifehzadeh, Fatemeh Dargahian, Samira Zandifar

**Affiliations:** 1 Desert Research Division, Research Institute of Forests and Rangelands, AREEO, Tehran, Iran; 2 Range Research Division, Research Institute of Forests and Rangelands, AREEO, Tehran, Iran

**Keywords:** Ecology, Environmental sciences, Natural hazards, Solid Earth sciences

## Abstract

Considering the presence of 274 dusty days in 2021 in Zabol city, Iran, the present study aimed to evaluate the feasibility of using treated sewage effluent (TSE) for dust mitigation with natural methods of increasing land cover. Hence, first of all, the identification of sewage treatment facilities along with the volume and chemical status was carried out and compared to the various national and international legislation. Then, field investigation on land use and land cover, along with literature review on dust origins, sand detachment areas, and sand corridors in the study area will be assisted for optimal area suggestion. Note that, in the present study it was assumed that the application of TSE for wetting the surface to vegetation restoration resulted in wind erosion control in critical foci. The results showed that, so far, a total of 39,000 m^3^/day could be treated, in the whole study area. The TSE volume calculated based on two scenarios consisting, (1) data obtained from the related organization, and (2) based the capacity of the wastewater plant is 2.8 and 5.1 mcm/year, respectively. Additionally, the study of TSE quality and its comparison to various regulation such as FAO, USEPA, INS, and CWQI indicated the applicability of transforming TSE to 14 km away from the WWT planet daily for rehabilitation of Hammon Hirmand through irrigation of *T.stricta* to increase the vegetation cover to above 30%.

## Introduction

Wind erosion is causing desertification and land degradation due to the movement of fine soil particles to water bodies, air, and other lands, increasing the risk to human health^1,2^, changing the soil properties such as structure, moisture content, and organic matter^3^ in arid and semiarid regions. It is the dominant problem in about 907,293 km^2^ of the deserts of Iran^4^. Various practices have been introduced to prevent or reduce soil erosion by wind consisting of roughening the soil surface, increasing the percentage of non-erodible clods, reducing field length, establishing and maintaining vegetative cover, and using wind barriers^5^. Wetting the surface to temporarily increase the percentage of non-erodible clods and establishing and maintaining vegetative cover are the most natural and effective methods^6,7^.

Due to the water resource limitations in arid and semi-arid regions, the feasibility of applying treated sewage effluent (TSE) (also commonly known as reclaimed water, recycled water, or reused water) was previously studied^8^. It generally has been applied for irrigation^9^, groundwater replenishment^10,11^, industrial processes^11^, and environmental restoration^12,13^. However, other applications for rangelands^14^, forests^15^, recreation areas, including parks and golf courses^16^, and disturbed lands^17^, such as mine spoil sites^18,19^ were also investigated. The TSE implementation for dust control or surface cleaning of roads, construction sites, and other trafficked areas^20^.

According to the reliable authorities, TSE is the most important and practical form of using unconventional sources, due to the stability discharge throughout the year and the possibility of planning and considering infrastructure. TSE can be applied to tree plantations after receiving only primary treatment with a low-cost lagoon primary treatment system and hence is beneficial in reducing fertilizing costs, as it is typically rich in organic matter, nitrogen, phosphorous, and several plant micronutrients^21,22^. Hence, the organic amendments which have been used to fertilize soils^23–25^ can be replaced by either the biosolids or even the effluent.

Despite the approval of the EPA and state environmental agencies, concerns exist regarding the potential survival and transmission of pathogenic microbes derived from biosolids after they applied to agricultural land^26–28^. Therefore, the microbial statements through the quality measurements of TSE before its application is inevitable.

According to the above explanations, the present study aims to study the feasibility of using TSE in dust reduction/mitigation through the rehabilitation of Hamuns lake near the sand movements and dust hotspots (corridors) that causes damage to some vital access roads of the study area in Sistan province, Iran. The aim of the present study is firstly to evaluate the feasibility of using TSE based on its quality and quantity. Comparing the results with various international and national standards has been used. Later, for the rehabilitation and development of Hamuns lake in the study area, the best matchable regions that could stop the dust emission based on the priority were demonstrated.

## Material and methods

### Study area

**Figure 1:**
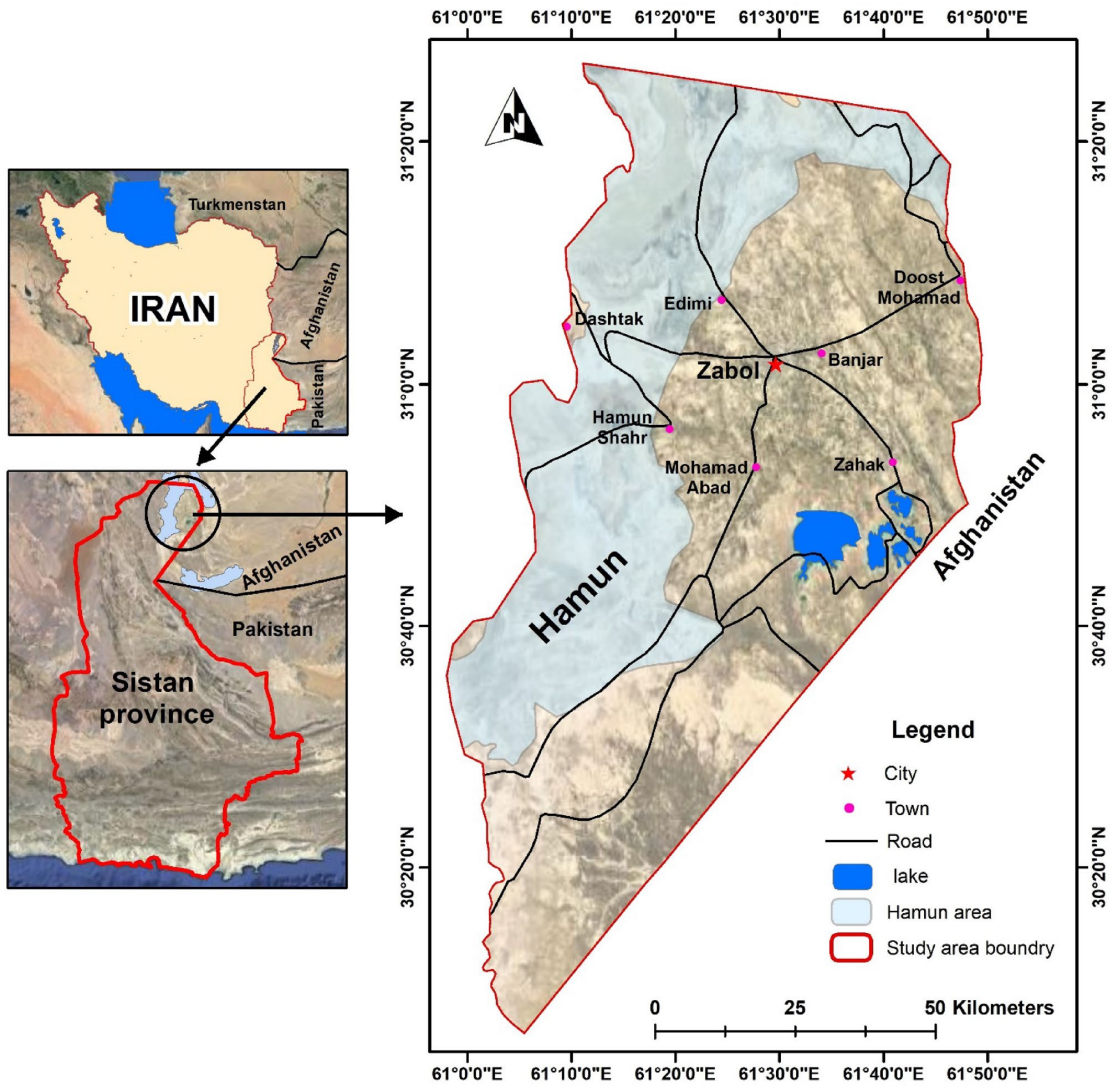
Location of the study area in the country, along with the cities of the region © 2022 by Springer Nature Limited is licensed under Attribution 4.0 International (created by ArcMap 10.5).

The study area is Zabol city located in the Hamun-Hirmand watershed, in Sistan Province (30° 5′ N–31° 28′ N and 61° 15′ E–61° 50′ E) close to the Iranian border with Pakistan and Afghanistan, in the southeastern part of Iran (Fig. 1). Sistan region has a population of 400,000 in six cities of Zabol, Benjar, Zahak, Mohammadabad, Edimi, and Dost Mohammad, and 980 villages. The Zabol synoptic station showed an arid climate with an average precipitation of 55 mm/year while evaporation exceeded 4000 mm/year^29^.

It was stated that the dust problem in Sistan caused by the specific wind regime^30^ and folded by the mismanagement of water resources. During the summer season, the area is under the influence of a low-pressure system considered as the trigger for the development of the Levar northerly wind, known as the ‘‘120-day wind’’^31^, causing frequent dust and sand storms and reducing of air quality, especially during the summer (June to August)^32^.

Continuously, droughts over the past decades^29^ impacted the Hirmand river, the main watershed for the Sistan basin, where agricultural croplands are irrigated. Additionally, it has caused the desiccation of the natural swamp of the Hamun lake complex (e.g., Hamun Saburi, Hamun Puzak) in the north of the Sistan region (Fig. 1), as the river finally drains into them. Hence, the powerful ‘‘120-day wind’’ effortlessly lifted fine sands of the exposed lake bed, making the basin one of the most active sources of dust^32^ and deposit within huge dune bed forms along the lakeshore. Consequently, the defective circulation negatively impacted the social society, cities, villages, structures, health and environment.

**Figure 2:**
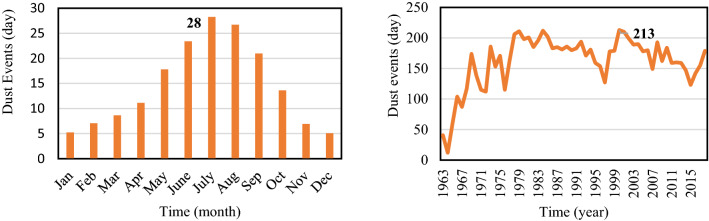
Dust events in the time scale of month and year in the study area.

Zabol had 9106 dusty days in the period of 1965–2018. Based on the results of dusty days as showed in Fig. 2, the average of 28 days for July and 213 days on 2000 recorded as the most.

**Figure 3:**
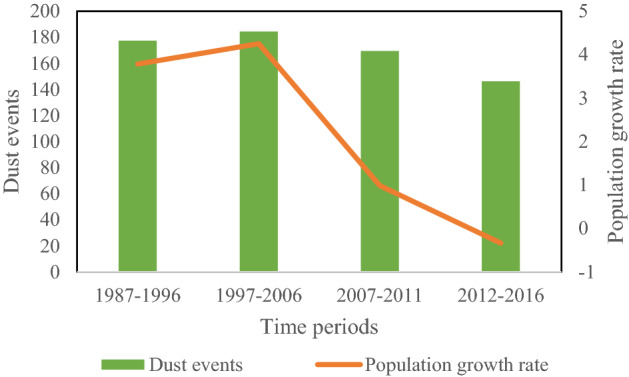
The comparison of population growth rate and average of dust days in the study area in the period of 1965–2016.

Moreover, the investigation of the population growth rate and dust events in the study area (Zabol) in the period 1965–2016, indicated a very strong correlation (Fig. 3). The decreasing trend can be explained by the socio-economic impacts of dust on agriculture and the high rate of immigration and fertility; in which the importance of dust reduction in the study area is arguable. Among various natural, chemical, biological and physical stabilization methods, the present study focused on the application of discharged TSE for dust reduction.

### Data source

The data source in the present study is mainly composed of the volume and chemical characteristics of the sewage and TSE. The data were obtained from the Iran Department of Environment, Water and Wastewater Company of Iran, and the Water and Sewerage Department of Sistan Province, Iran.

### Sewage and TSE characteristics

In the present study, to estimate the sewage volume of urban communities, the appropriate coefficient of sewage in the study area has been determined and applied to the water consumption. Based on regulation of the Department of Environment^33^ in Iran, the average per capita water consumption in urban communities is between 150 and 220, and in rural areas between 80 and 120 L/day. It should be noted that the above numbers are typical and, in some urban, and rural regions the amount of water consumption may be more or less than the mentioned range. Per capita, sewage production is the amount of sewage produced by each person per day. To calculate the per capita sewage production from the amount of water consumption, a coefficient is defined as “the coefficient of conversion” of water into sewage between 70 and 90% depending on the life style and climatic conditions^34^. Also, the main factor in determining the percentage of sewage returned to surface and groundwater sources called “the coefficient of connection” to the sewage collection network. Hence, in the study area of Zabol, the sewage coefficient is equal to 72%; while the percentage of return to underground and surface resources is 70 and 30, respectively.

Since in none of the studied villages, there are no sewage collection and treatment facilities in operation, so it can be said that the major percentage of sewage enters groundwater resources and rarely (in areas with steep slopes) enters surface water sources.

Furthermore, the existing industrial sewage treatment plants in the study area has the input of 50 m^3^/day. Low volume of industrial sewage compared to urban sewage, high pollution load, and the existence of points of this unconventional water source are among the features that need to take special measures to manage its reuse. Hence, it is not considered in the present study.

The produced sewage and TSE were calculated by assuming that after the upgrade of the sewage treatment plants, the TSE can be used for dust mitigation. The calculation of the water quantity of the TSE supply is as showed in Eq. (1), where S represents the amount of TSE (m^3^) that can be supplied by the sewage treatment plant, *Ci* represents the daily processing capacity of the sewage treatment plant (m^3^/day), *Ri* represents the number of days sewage treatment plant *i* is operational during 1 year (days), and *n* represents the number of sewage treatment plants with water quality standards^35^.

1 $$S={\sum }_{i=1}^{n}\mathrm{Ci}\times \mathrm{Ri}$$

### Sewage and TSE quality characteristics

One of the aims of this study was to identify and improve environmental safety by compare the quality of TSE with various standards including USEPA, CWQI (Canadian water quality index) and IDE (Iran department of environment), by ensuring the quality and quantity of the TSE. Note that the reclaimed water principles for development of the idea of using TSE for the dust mitigation is to be harmless for direct human contact after sewage treatment.

### Potential application of TSE

When unconventional water sources planned to be used instead of good quality water sources in various applications, the chemical parameters must have been testified for ensuring its effects on the environment. Therefore, in the present study, after the quality studies of the TSE, the evaluation on the application was comprehensively carried out according to various national and international standards. Through the regulations, as TSE applications consisting of drinking, aquatic, irrigation, recreation, and livestock, was defined. TSE is frequently applied for irrigating of agricultural lands. However, in the present study, the feasibility on the application of TSE for rehabilitation of native plants of the active bed of Hamun Hirmand lake were investigated through wetting the surface supposing its impact on dust reduction/mitigation. As none of the categories was set for this study; therefore, the recreation and irrigation were found the best fit. Nevertheless, considering the safety factor in environmental engineering, the irrigation standards were also reported. Noting that the sensitivity is at a lower place in comparison to irrigation standards.

The comparison between the quality of the TSE and sewage is based on various regulations, including the food and agriculture organization (FAO), US environmental protection agency (USEPA), the Canadian water quality index (CWQI), and Iran’s national standards (INS) considering the irrigation and recreational application.

### Optimal area suggestion for project execution

Important natural ways to control dust are classified as preservation and restoration of forests and pastures with the participation of the local communities, and related organizations, watersheds, and aquifer management operations, creating and maintaining green space belts around cities, and the expansion of urban green space. Hence, firstly the volume and chemical status of the TSE was investigated and compared to the various national and international regulations. Later, the land use and land cover^36^, along with dust origins^29^, sand detachment areas^31^, and sand corridors^37^ in the study area will be assisted for determining the optimal area suggestion for project implementation. It is worth mentioning that the vegetation cover map was created using ArcMap 10.5. Note that after recording points of GPS device through field investigations, the points were updated and checked by the Iran’s ecological regions recognition map, along with the habitat factors, and satellites images^36^. The canopy percentage of different areas was evaluated using the linear transect method for evaluating bush and shrub plants in the study area^36^.

**Figure 4:**
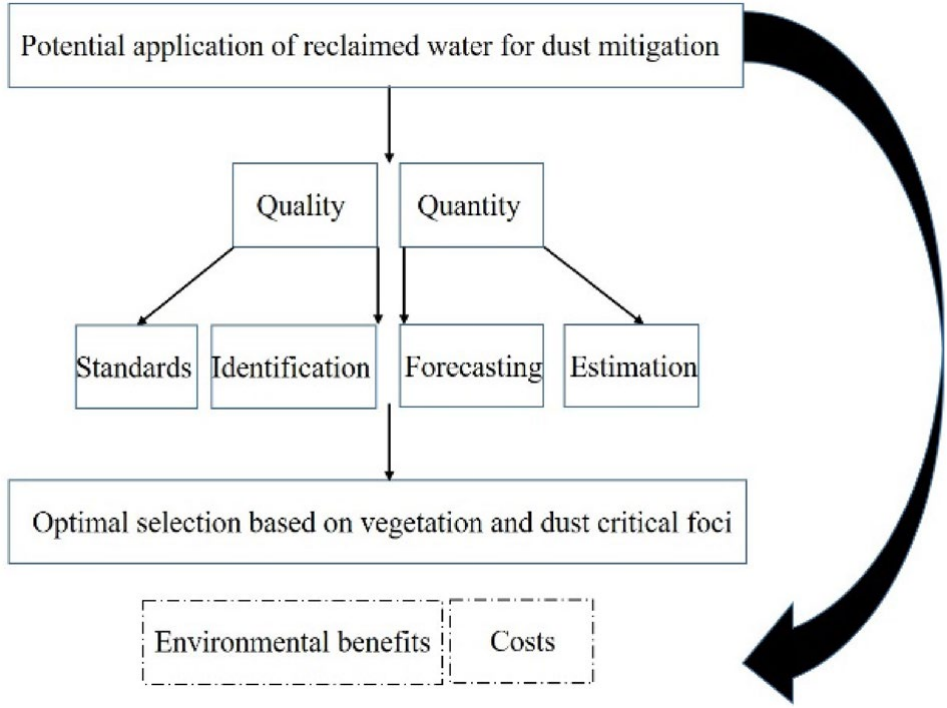
Outline of the conceptual model of methodology.

Note that, in the present study it was assumed that the application of TSE for wetting the surface to vegetation restoration resulted in wind erosion control in critical foci of Sistan. Accordingly, the most promising areas for TSE implementation to control dust will be suggested. The methodology is illustrated in Fig. 4.

## Results and discussion

### Sewage and TSE quantity characteristics

The WWT facilities have been implemented for Zabol with a capacity of 39,000 m^3^/day. Table 1 shows the volume of water consumption and sewage production based on the sewage coefficient in urban communities of the study area.

**Table 1:** Water consumption, TSE volume and receiving resources in the study area—2019.

Name	Water consumption (mcm/year)	Sewage coefficient	Sewage volume (mcm/year)	Percentage of return on resources		Sewage input volume (mcm/year)	TSE volume (Scenario 1) (mcm/year)	TSE volume (Scenario 2) (mcm/year)
				Underground	Surface			
Zabol	22.538	0.72	16.194	70	30	4.86	2.81	5.1

As shown in Table 1, the total water consumption in the study area is 22.538 mcm/year while based on the development conditions. Afterward, the sewage volume was calculated to 16.194 mcm/year, considering the sewage coefficient and water consumption.

Continuously, the sewage data obtained from the Water and Wastewater Organization of Zabol city, Iran, showed that the sewage entrance to the treatment plants of the study area is about 19,000 m^3^/day and 137 working days. Therefore, the TSE volume of the WWT plant was calculated based on the following scenarios of (1) data obtained from the Water and Wastewater Organization, Iran, and (2) based on the capacity of WWT plant. Note that the working days for both scenarios will be 137. The calculation is based on Eq. (1). The total TSE volume for scenarios 1 and 2 is 2.8 and 5.1 mcm/year, respectively.

The difference between the calculation based on capacity and the existing data is due to the removal of raw sewage before entering the treatment plant, which has caused health and environmental problems in the region. Data obtained from Iran Department of Environment^34^ showed that 1.68 mcm/y of sewage were extracted for the farms. Previous studies in the same study area also reported the significant (P < 0.05) value of heavy metals in the soil of agricultural lands due to the application of sewage instead of TSE^38^. The results also revealed the efficiency of the WWT facilities based on scenarios 1 and 2, so far, very little of the potential of TSE resources in the study area was executed and a total of -90 percent of the treatment is underused. However, it is necessary to mention that more working days of WWT plant, could not increase the TSE volume, as the sewage volume based on return percentage is still low and around 30%. Thus, it is recommended that the government or private sector invested on the raising the coefficient of return.

### Sewage and TSE quality characteristics

Water quality is the key factor in TSE reuse. Currently, Iran’s urban sewage treatment process mainly involves conventional methods such as coagulation, sedimentation, filtration, and disinfection. The methods of sewage treatment in Zabol and Zahak, in the study area, are stabilization pond and oxidation pond, respectively. In other urban areas, the produced sewage enters directly into the absorption wells of sewage disposal or inappropriately enters the environment. The stabilization pond is practical in hot and sunny areas because sunlight causes the photosynthetic action of oxygen to provide the oxidation required by sewage pollution. In addition, in the treatment method of stabilization ponds, no wastes that need special treatment are produced, and the quality after that is among the latest treatment methods of sewage quality and used in irrigation of agricultural lands, parks, and surface waters.

The chemical characteristics of sewage and TSE was examined on a seasonally and yearly scale. The average values of physicochemical factors in sewage and TSE of the Zabol sewage treatment plant in three months of summer are presented in Table 2.

**Table 2:** Seasonably values of physicochemical factors of Zabol sewage and TSE (mg/l).

	pH	EC	DO	TSS	TDS	BOD	COD	TH	TKN	NO3	NO2	Ni	Co	Cr	Fe	Zn	Cu	Mn	Pb	Li
Sewage	8.2	14.06	1.98	82.64	38.96	131	280	475	1.81	0.89	0.021	0.076	0.06	0.01	2.25	1.33	1.22	3.47	0.95	0.14
TSE	7.94	1325	1.83	67.98	32.04	91.85	192.4	459	1.7	0.23	0.02	0.05	0.01	0.001	0.6	0.32	0.32	0.94	0.29	0.13

**Figure 5:**
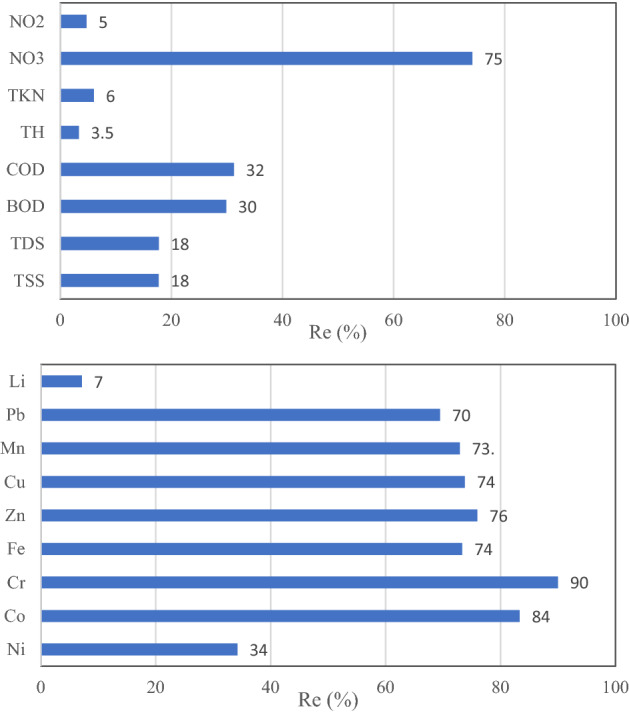
The efficiency of average seasonally values of WWT facilities in the study area.

As shown in Fig. 5, the efficiency of average seasonally values of input and output of the WWT facilities in the study area indicated that the highest removal efficiency is achieved for nitrate equal to 75% while the lowest was for TH with 3%. On the other hand, among the heavy metals and trace elements, the highest and lowest removal efficiency is for Cr and Li, respectively.

**Figure 6:**
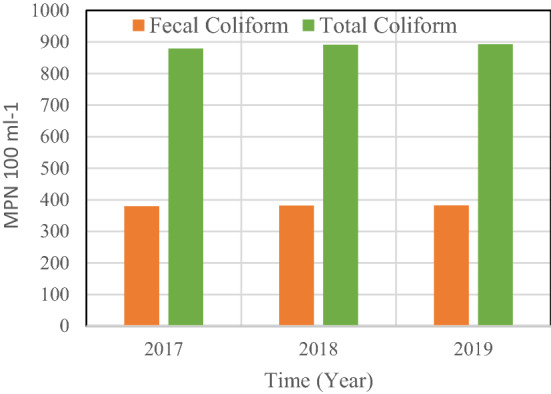
The results of the abundance of total coliforms (TC) and fecal coliforms (FC).

Continuously, bacterial indicators of fecal contamination and enteric viruses are present in high concentrations in raw sewage. Hence, the values of TSE in the period 2017–2019 of the Zabol WWT plant showed in Fig. 6. The results of the fecal coliform (FC) removal efficiency showed that removal of culturable FC reduced by order to > 5. Note that typical abundance of total and fecal coliforms (FC) in raw sewage are 107–109 and 106–108 100/mL, respectively, and were reduced by 1–5 orders of magnitude in treated TSE, depending on the type of treatment^39,40^. Classical treatments, which do not include any specific disinfection step, reduce fecal micro-organisms densities by 1–3 orders of magnitude^40^, but because of their high abundance in raw sewage, they are still discharged in large numbers with treated TSEs in the environment.

**Figure 7:**
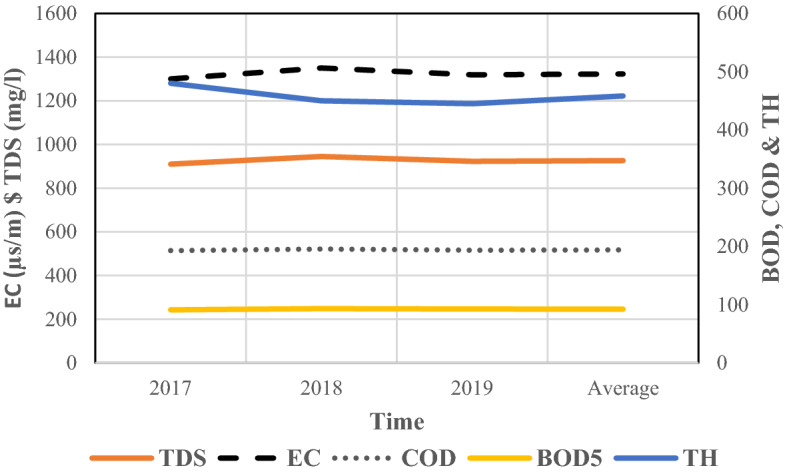
The results of yearly values of physicochemical factors of Zabol TSE.

Additionally, the results of yearly values of physicochemical factors of Zabol TSE (mg/L) including BOD5, COD, TDS, TH, and EC in the period of 2017–2019, showed in Fig. 7. The yearly results suggested that the values through the years of investigation did not show significant changes. In the following parts, the possibility of TSE evaluated considering various standards.

### Potential application of TSE

Comparing the quality of the TSE and sewage are based on various regulations showed in Table 3. It includes the food and agriculture organization (FAO), US environmental protection agency (USEPA), the Canadian water quality index (CWQI), and Iran’s national standards (INS), considering the irrigation and recreational application.

**Table 3:** Guidelines for interpretations of water quality of sewage and TSE of Zabol WWT plants (average in the period of 2017–2019) compared to the standards of regulations.

Parameters	TSS (mg/l)	TDS (mg/l)	pH	EC	SAR	Chlorine (mg/l)	BOD (mg/l)	COD (mg/l)	NO_3_^−^ (mg/l)	PH_4_^−^ (mg/l)	TC (MPN 100 ml)	FC (MPN 100 ml)
Sewage	190	980	8.1	1406	10	–	140	320	14.89	–	1341	914
TSE results	70	926	8	1300	6	0.3	94	193	7.7	13	893	382
FAO (Irrigation)	–	1750	6–9	2500	9	1	30	60	–	–	–	200
USEPA (Irrigation)	30	2000	6–8.5	3000	–	–	–	–	–	–	–	–
INS (Irrigation)	100	–	6.5–8	–	–	0.2	100	200	6	–	1000	400
INS (Recreation)	100	–		–		1			10	6	1000	400

According to the FAO Guide^41^ for Classifying Agricultural Water Quality, as shown in Table 3, the most crucial parameters for the application of TSE in irrigation include electrical conductivity (EC), sodium uptake ratio (SAR), chlorine, BOD, COD, and FC. However, three out of seven parameters namely BOD, COD, and FC in the TSE are largely erratic with the limits recommended in the standards.

Based on USEPA^42^, the value of total suspended solids in TSE of Zabol WWT plant largely inconsistent with the limits recommended in the standards for TSE reuse. However, TDS, EC, and pH, met the criteria. Moreover, except TSS and pH, the other chemical parameters of sewage also meet the criteria. It is worth mentioning that EPA does not require or restrict any types of water reuse. Generally, states maintain primary regulatory authority (i.e., primacy) in allocating and developing water resources. Some US states have established programs to specifically address reuse, and some have incorporated water reuse into their existing programs. EPA, states, tribes, and local governments implement programs under the Safe Drinking Water Act and the Clean Water Act to protect the quality of drinking water source waters, community drinking water, and waterbodies like rivers and lakes.

According to INS regulations for irrigation and recreation reuse of TSE^33^, the value parameters tested for the TSE of the Zabol WWT plant are following the limits recommended in the standards for consumption as irrigation (except chlorine) and recreation projects.

Finally, the CWQI is a means to provide consistent procedures for Canadian jurisdictions to report water quality information to both management and the public. The CWQI value ranges between 1 and 100, and the result is further simplified by assigning it to a descriptive category in Table 4.

**Table 4:** The CWQI value and descriptive.

Rank	WQI value	Description
Excellent	95–100	Water quality is intact; conditions are very close to natural or desired levels
Good	80–94	Water quality is intact; and only one minor threat or deterioration is observed, conditions rarely differed from the natural or desirable level
Fair	65–79	Water quality is usually intact, but occasionally endangered or deteriorated; conditions sometimes deviate from natural or desirable levels
Marginal	45–64	Water quality is frequently endangered or deteriorated. Conditions often deviate from natural or desirable levels
Poor	0–44	Water quality is always endangered or deteriorated; conditions usually deviate from natural or desirable lev

**Figure 8:**
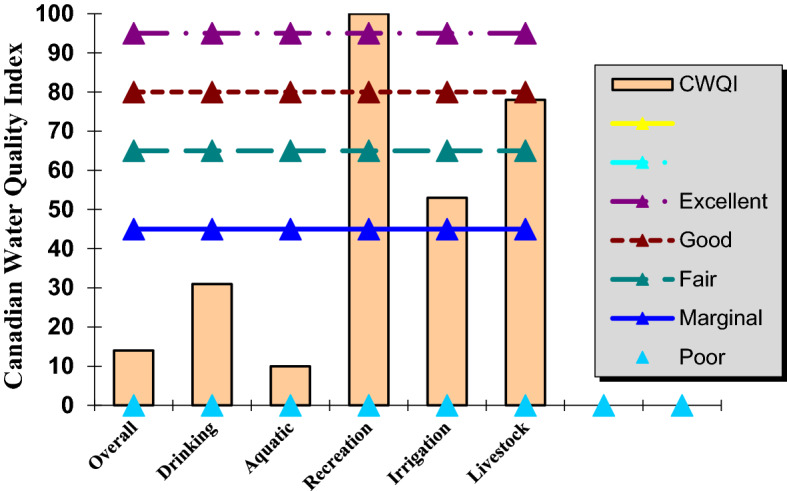
CWQI tets results for TSE of WWT plant in the study area.

The results of CWQI software for analyzing the TSE of the WWT plant in the study area, as shown in Table 5 and Fig. 8, indicated its poor quality for drinking, and aquatic. While it is fair for livestock and marginal for irrigation. However, considering the purpose of this study for irrigation of the native plants, it met the criteria. Note that the input data set is based on the period of 2017–2019.

**Table 5:** The results of TSE in various applications assessed by CWQI.

Data summary	Overall	Drinking	Aquatic	Recreation	Irrigation	Livestock
CWQI	14	31	10	100	53	78
Categorization	Poor	Poor	Poor	Excellent	Marginal	Fair
F1 (scope)	80	57	86	0	50	20
F2 (frequency)	80	57	86	0	50	20
F3 (amplitude)	98	88	98	0	42	25
Minimal dataset requirement of 4 variables	Met	Met	Met	**Not met**	Met	Met
Contaminant analysis of last sample	Passed	Not tested	Not tested	Not tested	Not tested	Not tested

Significant values are in bold.

The results of this section indicated the consideration of various parameters due to various regulations and demonstrated that the treatment technology upgrade was significantly better than those of urban miscellaneous water and agriculture water standards, indicating this system can be widely used for urban landscape hydration. Moreover, squeezing the sewage treatment process for being cost effective could be recommended considering the measurements of FC, BOD, and COD.

### Optimal area suggestion for project execution

**Figure 9:**
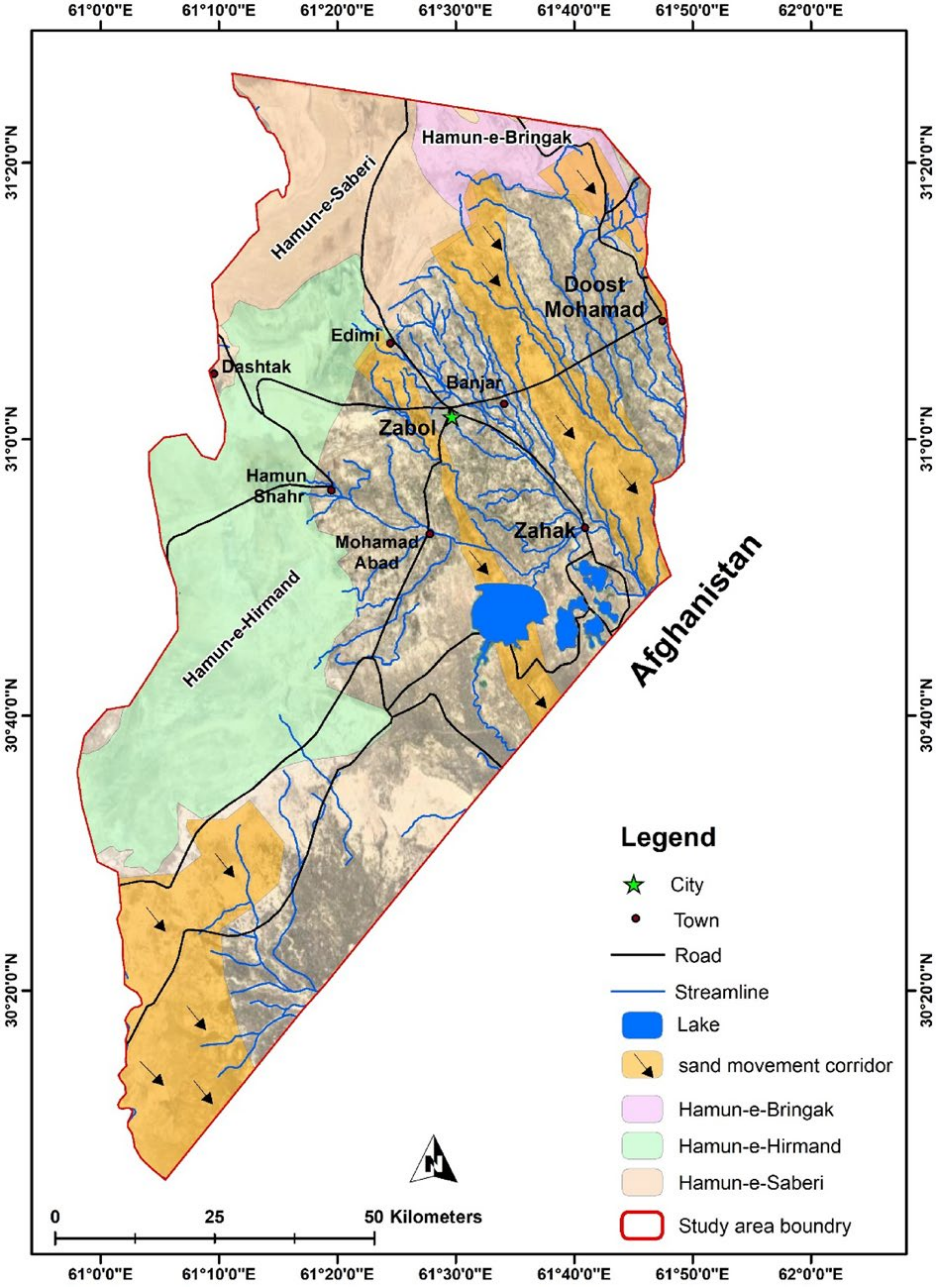
Locations and names of Hamuns lake and sand movement corridors in the study area © 2022 by Springer Nature Limited is licensed under Attribution 4.0 International (created by ArcMap 10.5).

Considering three steps of wind erosion which are detachment, transportation, and deposition, the sand fixation methods have to be done in the detachment area to be more effective. Hence, the most advantageous regions for project execution were selected based on the factors of (a) discovering the dust origins, and (b) vegetation cover. Regarding the first concern, it was shown that the dry sediments of the Farah river^43^, and the presence of dunes between the two sand movements corridors in Sistan, namely Jazinak (near Zabol city) and Tasuki corridors (shown in Fig. 9), was increased the dust concentration in Zabol city^37,44^ while the agricultural lands, and other infrastructures such as roads, and irrigation canals developed in the area between Zahedan and Zabol city.

**Figure 10:**
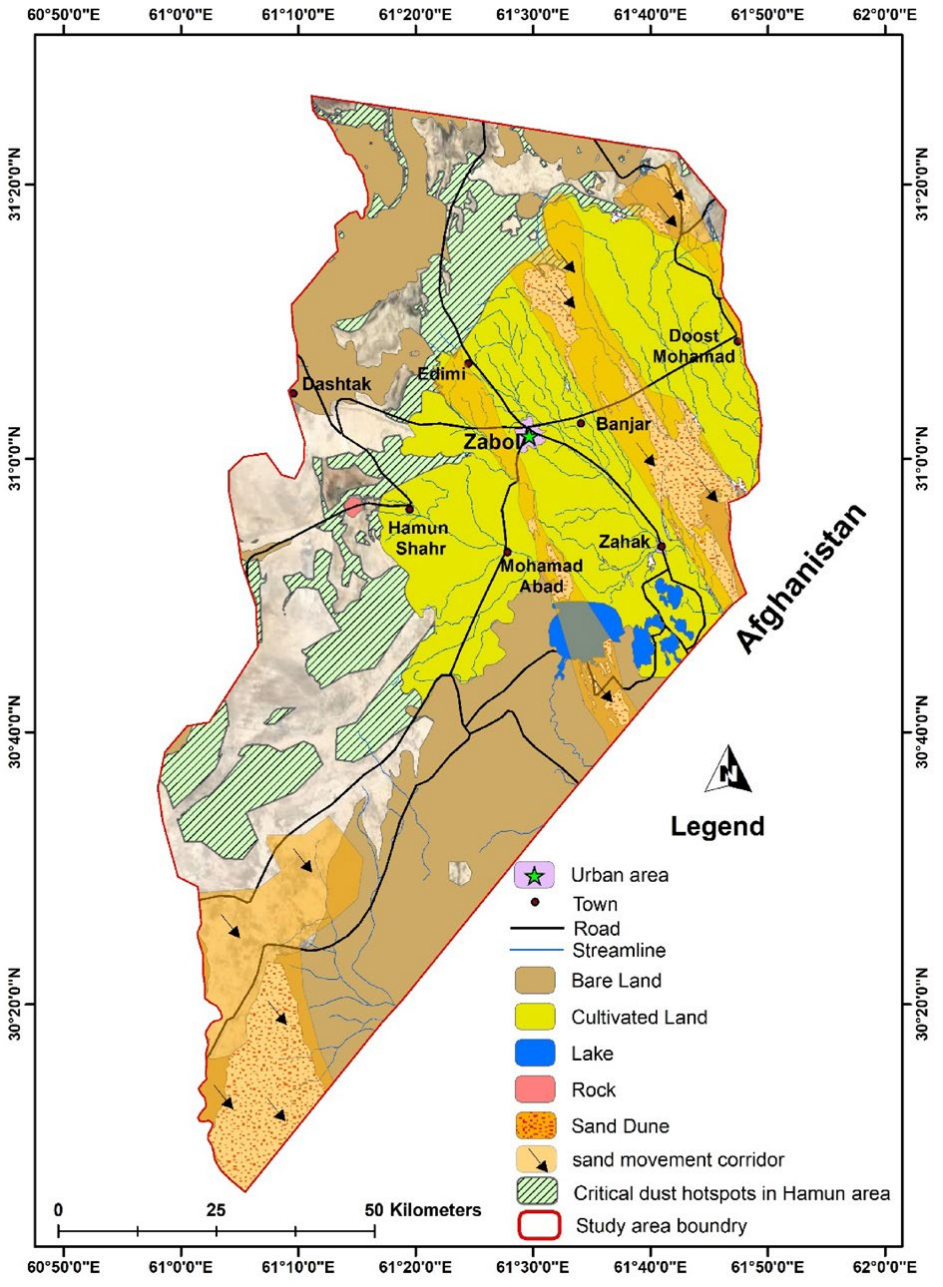
The critical dust hotspot and dust origins in the study area © 2022 by Springer Nature Limited is licensed under Attribution 4.0 International (created by ArcMap 10.5).

Subsequently, based on a guide that 30% of vegetation cover has a significant effect on the process of soil detachment^45,46^, and soil protection in the desert areas^47^, the regions with less than 30% vegetation cover in the study area based on field observation was investigated and showed in Fig. 10. Field observation demonstrated that most areas along with the Jazinak sand corridor and Zabol city have 1–15% and 15–30%^36^, which are in the priority for stabilization.

The results are consistent with Abbasi et al.^37^, reported that the Hamun Baringak Lake plays a crucial role in the aeolian mobilization of sediments in the Sistan region because of the hydrological droughts that led to the gradual decline of the wetland vegetation cover. Notably, Jahantigh^48^, in the same study area, reported that the average forage yield of *Aeluropus lagopoides* in Hamun Hirmand lake in the condition of the water inflow and during drought, was estimated to be 8869 and 173 kg/ha, respectively. It can be explained by the effect of water presence on plant production and cover. However, the average of bare soil of Hamun lake was estimated to be 7.5% and 84.2% in the two periods of water inflow and drought, respectively^48^. It indicated the impact of dusty days. Therefore, the mentioned areas with the vegetation cover below 30% prioritized for stabilization techniques to dust reduction or mitigation.

**Figure 11:**
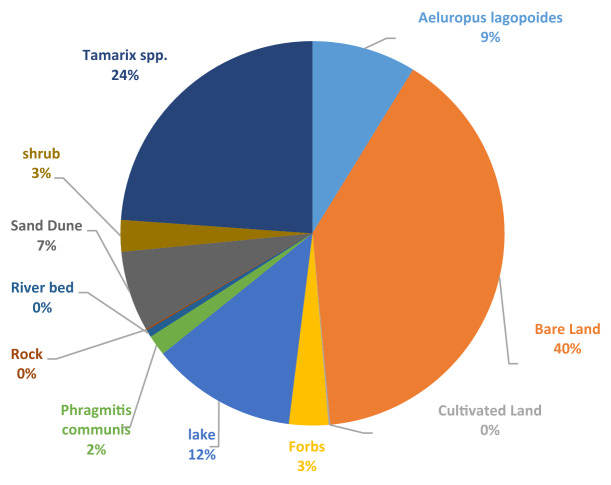
The most land use/cover in the study area.

**Figure 12:**
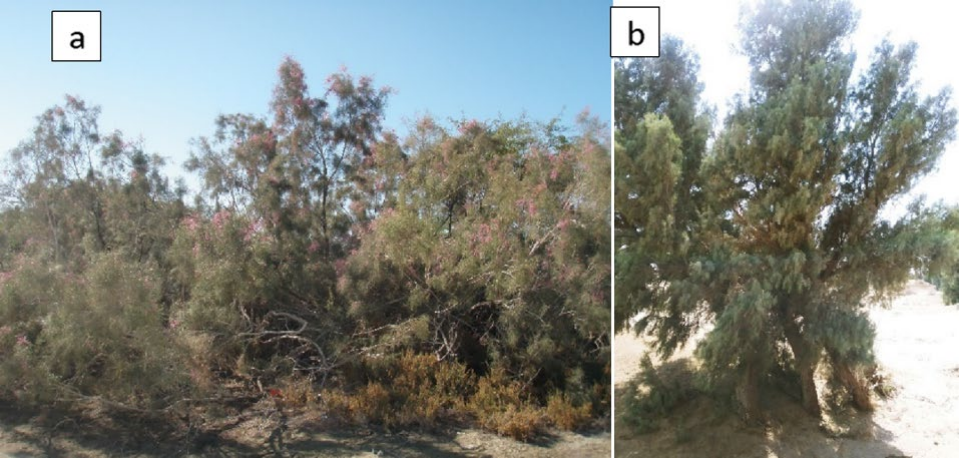
The picture of (**a**) *T. stricta* and (**b**) *T. aphylla* in the study area.

The detailed field investigation of the land use and vegetation cover, as shown in Fig. 12, indicated the presence of native plants such as *A. lagopoides* and *Tamarix* spp. Based on Fig. 11, among the *Tamarix* genus*,* the three species of *T. aphylla*, *T. stricta*, and *T.hispida* were observed in the study area. *T. stricta* is a native species to Iran with benefits including, traditional therapeutic uses in Persian Medicine^49,50^. Also, the soil EC in the habitat of *T. aphylla* (15.70 mhos/cm) is almost the same as the control area (15.80 mhos/cm) in the depth of 0–30 cm; while the available potassium in *T. aphylla* habitat (460 mg/l) was also more than the control area (180 mg/l)^51^. Hence, the afforestation of *Tamarix* spp. has caused the addition of soil amendments and increased the clods.

Consequently, the water requirement of the plants in the desert area consisting of *T.aphylla,* is reported in Table 6. The water requirement of *T. stricta* was estimated based on Table 6 to be 580 m^3^/ha for 500 plants no./ha with a vegetation cover of 10–30%.

**Table 6:** Annual water requirement of the *T. aphylla* for irrigation in the early stages of establishment in terms of planting density *(Rad, 2018).*

Plant species	Average of annual evapotranspiration (m^3^/ha)	Plant coefficient (Kc)	Coverage (%)	Density (no. per ha)	Annual water requirement (m^3^/ha)
*T. aphylla*	10,800	0.58	30–50	500–830	540–896

Moreover, Fig. 12 shows the vast (50% more) soil coverage of *T. stricta* in the collar area compared to *T. aphylla.* Therefore, it is more appropriate to cultivate *T. stricta* than *T. aphylla* for the biological restoration of the region. Note that the introduced dust mitigation technique using TSE of Zabol WWT can play a specific role in the rehabilitation of soil cover in the mentioned area due to the low water need of native plants. Consequently, it has a significant impact on dust reduction in Zabol city.

**Figure 13:**
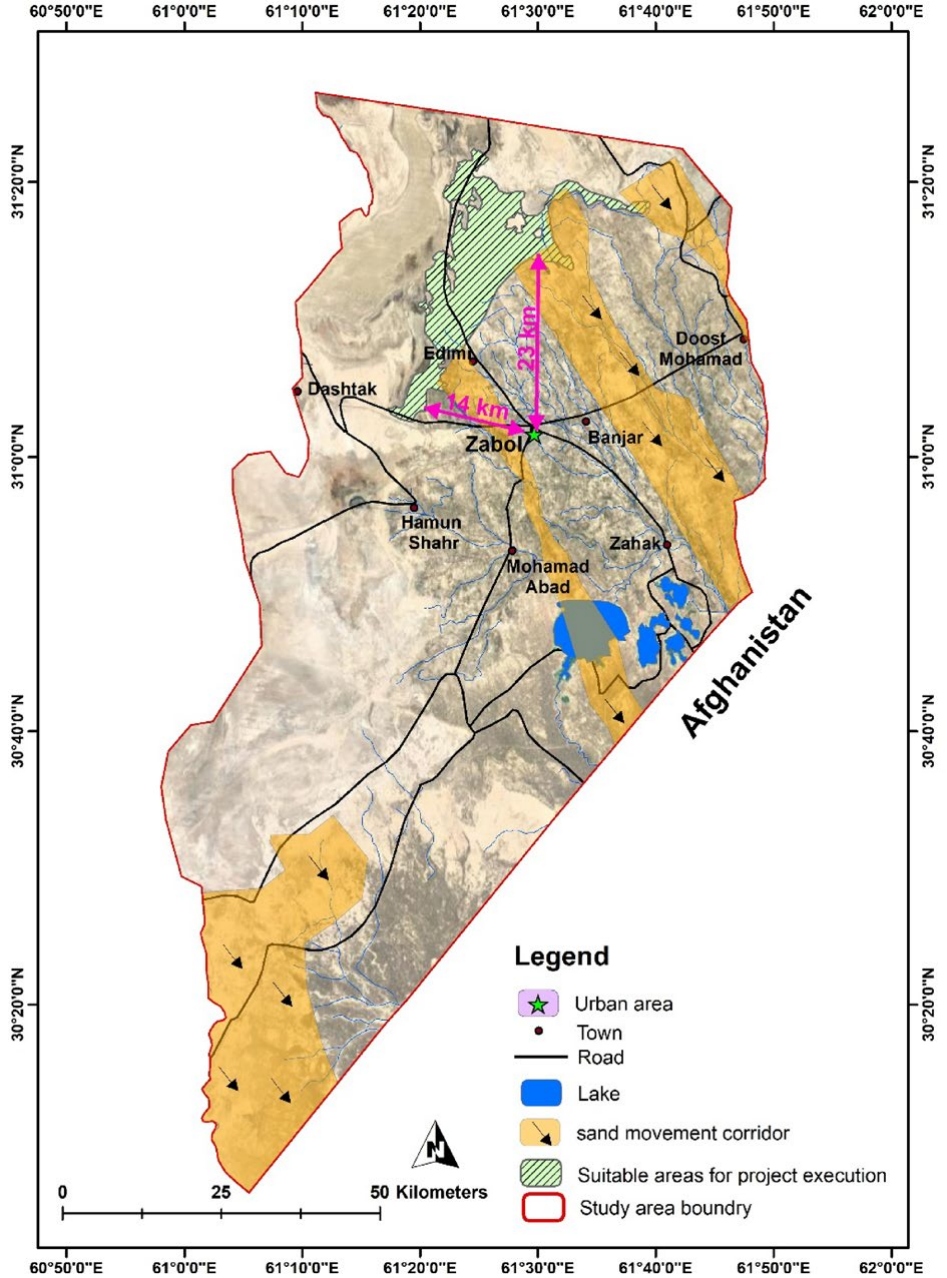
Area suggested for the dust mitigation project execution by the application of TSE © 2022 by Springer Nature Limited is licensed under Attribution 4.0 International (created by ArcMap 10.5).

Hence, based on the hotspots of dust origins in the study area, the most appropriate sites for the project executions of TSE were selected, as shown in Fig. 13. Investigations indicated that a total of 27,500 ha are suitable for the project excision. Hence, considering the water requirement of 500 m^3^/ha/year, TSE volume of 5.1 mcm/year, vegetation cover of below 30%, and other observations such as the soil coverage in the collar area, the native plant of *T. stricta* selected for the afforestation of 10,000 ha on the west part of Zabol. This region has the priority in stabilization due to companionship to the corridors with a vegetation cover of 16–30%.

### Cost analysis

Finally, due to the vast area of TSE application, the total of 27,500 ha, with the puprose of dust mitigation, the project execution costs must have been addressed. Hence, Fig. 13 shows the distance of Zabol city to Hamun Hirmand and Baringak lake for transportation calculation. Accordingly, the distance from Zabol to Hamun Hirmand and Baringak lake is 14 and 33 km, respectively. The whole area around Zabol city to Hammon Hirmand lake is cultivated lands; hence, the existing roads reduced construction costs.

The two main modes of transportation are trucks and pipelines. There are various pros and cons to both methods. Truck transportation is favored for low volume and short distances, while its costs rapidly increase for large-scale transportation. On the other hand, pipeline transportation is appropriate for large volumes, and long travel distances as it has a positive impact on reducing greenhouse gas emissions. Using pipelines also reduces noise, reduces highway traffic, and improves highway safety.

Based on the literature, the variable and fixed transportation cost components depend on the type of product shipped, design requirements, and other decisions related to facility planning. For the sewage sludge with a pH level of 7.0 ± 0.1; hence, a low-cost PVC pipe suggested. Moreover, for cost optimization, as the WWT facilities in the study area do not generate enough volume daily, it makes economical sense to store sewage for a few days to increase the shipped volume. However, reducing the storage to a single day condenses these investment costs drastically^52^.

It was estimated that the total costs for a facility-owned and rented single trailer truck with a capacity of 30 m^3^ to be $5.6/m^3^ and 7.4/m^3^/km, respectively^53^. Hence, the variable unit transportation cost along a pipeline with a capacity of 480 m^3^/day is estimated to be $0.144/m^3^/km. In despite of previous studies mentioning that it is more economical to use a pipeline rather than a rented single trailer truck if the volume shipped is greater than 700 m^3^/day, in the study area, it is more economical to use a facility-owned single trailer truck, while the shipped volume is 1200 m^3^/day due to the low cost of petroleum and very close distance of the suggested area.

## Conclusion

TSEs are a modified water source in the current drought and critical condition of alluvial aquifers. Therefore, in the present study, the feasibility of TSE application for dust reduction/mitigation, based on natural methods for Hammon lakes rehabilitation in southeast Iran, was investigated. Comparing the TSE chemical characteristics with various standards indicated that it met the criteria of the purpose of study for dust reduction/mitigation through soil wetting and increasing the native vegetation. It was shown that the afforestation by the introduced TSE of Zabol WWT plant on the north (Hamun Baringak lake) and west (Hamun Hirmand lake) parts of the study area play a vital role in the rehabilitation of soil cover in the mentioned area due to the low water need of native vegetation such as *T. stricta*. As a consequence, it has a significant impact on dust reduction in Zabol city due to the increase of the vegetation cover to above 30%. Finally, in the study area, it is more economical to use a facility-owned single trailer truck, while the volume shipped is 1200 m^3^/day due to the low cost of petroleum and very close distance of the suggested area.

## Data Availability

The datasets used and/or analyzed during the current study available from the corresponding author on reasonable request.
